# RPE disruption and hyper-transmission are early signs of secondary CNV with punctate inner choroidopathy in structure-OCT

**DOI:** 10.1186/s12886-021-02197-7

**Published:** 2021-12-10

**Authors:** Yanru Chen, Qian Chen, Xiaoxin Li, Minghan Li

**Affiliations:** 1 Xiamen University affiliated Xiamen Eye Center, Wutong West Road 989, Hu Li District, Xiamen, 361100 Fujian China; 2 Eye Institute of Xiamen University; School of Medicine, Xiamen University, Xiamen, 361102 Fujian China

**Keywords:** Punctuate inner choroidopathy, Choroidal neovascularization, Optical coherence tomography, Transmission

## Abstract

**Purpose:**

To study whether retinal pigment epithelium (RPE) disruption and choroidal hyper-transmission on spectral-domain optical coherence tomography (SD-OCT) are signs of inflammatory neovascularization (CNV) in punctate inner choroidopathy (PIC).

**Methods:**

This is a prospective cohort study. Seventeen patients (18 eyes) were diagnosed as PIC without CNV at baseline. Changes of morphological characteristics including choroidal hyper-transmission, hypo-transmission, RPE disruption, and ellipsoid zone (EZ) damage on SD-OCT were observed and recorded at baseline, 4, 8 and 12 weeks, respectively. The occurrence of CNV was detected by OCTA at each visit. Fisher’s exact test was used to compare the relationship with each morphological sign and evaluate the predictable capability of secondary CNV in PIC (PIC+CNV) based on the structure changes on OCT.

**Results:**

Among the 18 eyes, a total of 5 eyes (27.8%) developed PIC+CNV subsequently within 4 weeks follow-up. At 4, 8 and 12 weeks of follow-up, RPE disruption and choroidal hyper-transmission were found in all 5 PIC+CNV eyes. The incidence of RPE disruption was significant higher in PIC+CNV eyes compared with PIC eyes (*P*=0.001). PIC eyes with hyper-transmission had a higher risk for developing CNV compared with those without hyper-transmission (*P*=1.17×10^-3^). 2 out of 5 PIC+CNV eyes had a choroidal hypo-transmission component adjacent to hyper-transmission zone at 4 weeks of follow-up, and hypo-transmission could be observed in all 5 PIC+CNV eyes at 8 weeks of follow-up. The incidence of choroidal hypo-transmission was significant higher in PIC+CNV eyes than PIC eyes after 8 weeks. EZ damage began to recover at 4 weeks of follow-up and had no significant difference in the PIC eyes and PIC+CNV eyes (*P*=0.150, 0.196, 0.353).

**Conclusion:**

The presence of choroidal hyper-transmission and RPE disruption on SD-OCT is associated with the PIC+CNV. SD-OCT imaging facilitates the differentiation and track of the progression of inflammatory lesions and secondary CNV in PIC.

## Introduction

Punctuate inner choroidopathy (PIC) is an ocular inflammatory disease mostly affecting young myopic women. Patients with PIC present symptoms of loss of central visual acuity (VA), metamorphopsia and scotomata. Although PIC is usually a benign disease; complications such as inflammation, choroidal neovascularization (CNV) and subretinal fibrosis can lead to severe vision loss [1]. Depending on the course of the disease and development of complications, treatment may range from observation to immunomodulatory therapy and/or anti-vascular endothelial growth factor (VEGF) therapy. Inflammation may play a pathogenic role in PIC; while choroidal neovascularization is a common complication as the disease progressed [2]. However, hyper-fluorescence and leakage can be found in both active inflammatory lesions and CNV, it may be difficult to differentiate them by fundus fluorescein angiography (FFA). The newly developed optical coherence tomography angiography (OCTA) provides abnormal blood flow signals, which can aid in the differential diagnosis of choroidal neovascularization and inflammation [3]. But it is not widely used than optical coherence tomography (OCT) in rural and community hospitals in developing countries [4]. To easily distinguish the PIC inflammation lesion and secondary CNV, we utilized spectral domain OCT (SD-OCT) to study the morphological characteristics of these lesions. It can detect the morphological changes of different layers of the retina and choroid with high sensitivity, specificity and depth resolution. Thus, SD-OCT is a good tool to study PIC, which usually affects the level of the deep retina and choroid.

## Methods

### Patients

Seventeen patients (18 eyes) with PIC who complained acute blurring of vision within 4 weeks from Xiamen Eye Center from April 2019 to April 2020 were included in the prospective study. The inclusion criteria were as follows: (1) Presence of one or several yellow, white or gray spots (diameter most ≤ 500μm) mostly limited to the posterior pole at the level of retinal pigment epithelium (RPE) and inner choroid with minimal vitreous reaction; (2) SD-OCT imaging revealed lesions at the level of the deep retina and choroid; (3) SD-OCTA showed no evidence of CNV. Exclusion criteria were as follows: (1) Eye diseases such as retinal vascular occlusion, diabetic retinopathy, age-related macular degeneration, optic nerve diseases, and glaucoma that affect vision; (2) Any other type of CNV; (3) After any treatment such as anti-VEGF, glucocorticoid; (4) Other white dot syndromes, such as diffuse subretinal fibrosis, acute posterior multifocal placoid pigment epitheliopathy, serpiginous choroiditis. This study was conducted under the tenets of the Declaration of Helsinki and was approved by the ethics board of Xiamen University affiliated Xiamen Eye Center, Xiamen, China. Written informed consent was obtained from all patients. All patients provided informed consent after a thorough description of the nature and consequences of the study.

### Clinical examinations

Each patient underwent best corrected visual acuity (BCVA) measurement using a Snellen chart, and BCVA was then converted to logarithmic minimal angle of resolution (logMAR) units for statistical analysis. Slit lamp was used to check the anterior chamber. Dilated fundoscopy, fundus photography (KOWA nonmyd WX3D, Optos 200Tx) were applied to examine the retina. SD-OCT (Heidelberg Engineering, Heidelberg, Germany) was performed on the PIC eyes with enhanced depth imaging (EDI) and eye tracking function using a line scan, volume scan modes, which were across macular fovea and cover lesions. Enhanced depth imaging mode with deeper signal penetration providing enhanced identification of the choroidal vascular layers, and choroid sclera junction. The SD-OCT scanning protocol consisted of an A-scan through the center of the macula with a length between 8.8 mm and 10.0 mm in the horizontal and vertical direction and 19 B-scans covering an area of 20°×15.0° centered on the fovea with a scanning depth of 1.9mm. SD-OCTA (Cirrus HD-5000, Carl Zeiss, Germany) was performed in a 3 mm × 3 mm macular cube centered on the fovea and a 6 mm × 6 mm HD macular cube to encompass the lesions. Image analysis was performed by automated segmentation derived from the machine software with manual adjustments. OCTA images were acquired at the choroid, choriocapillaris, outer retina, deep capillary plexus, and superficial capillary plexus layers. Morphological characteristics including the presence of choroidal hyper-transmission, choroidal hypo-transmission, the RPE intact or disruption, and the ellipsoid zone (EZ) intact, partially continuous, or absent. These morphological characteristics were checked and compared at each visit. Patients with/without any morphological characteristic were classified into two groups. The changes of each morphological characteristic were observed and recorded at baseline, 4, 8 and 12 weeks, respectively. All of the patients were all preliminarily diagnosed as PIC without CNV at baseline as confirmed by OCTA. The occurrence of CNV was detected and confirmed by OCTA at each visit. In addition, the incidence of PIC+CNV was calculated for each patient if there is one or more morphological characteristics. The associations between morphological characteristics and CNV were analyzed. Two reviewers (CYR and LMH) separately assessed the images. Discrepancies in their findings were referred to a fundus specialist for a final determination.

### Statistical analysis

Statistical analyses were conducted using IBM SPSS Statistics version 21.0 (IBM Corp., Armonk, NY, USA). Normality of the variables was examined by Shapiro-Wilk normality test. The age, refractive error, and VA were normalized (*P*=0.278, *P*=0.243, *P*=0.401). The measurement data were expressed as Mean ± Standard Deviation; the enumeration data were expressed as a constituent count or percentage. The rate of each morphological sign on OCT and the incidence of PIC+CNV was compared by Fisher’s exact test, respectively. A P value of < 0.05 was considered statistically significant.

## Results

### Patients’ characteristics

A total of 17 patients (18 eyes) with PIC who complained of acute blurring of vision within 4 weeks were enrolled in the study. The mean age was 31.89±9.19 years old (ranging from 18 to 53 years old). Eleven (61.1%) of them were female. The mean refractive error was -10.47±4.38 diopter (ranging from -2.00D to -18.00D). The best corrected visual acuity (Log MAR) at baseline was 0.69±0.33. Choroidal neovascularization confirmed by SD-OCTA was developed in 5 eyes (27.8%) during follow-up. Thirteen (72.2%) patients without any evidence of CNV throughout the time of observation required no therapeutic intervention, as their inflammatory lesions resolved and their visual acuity improved; at the last visit, the average visual acuity was 0.31±0.20. All the patients with CNV received vascular endothelial growth factor inhibitor (anti-VEGF) treatment, and the CNV lesions resolved following with improved vision acuity.

### SD-OCT features

#### RPE and Bruch’ s membrane integrity

At baseline, 18 eyes had neither RPE nor Bruch’s membrane (BM) disruption, while RPE elevation were observed in 5 eyes. At 4 weeks follow-up, these 5 eyes had developed secondary CNV that was confirmed by OCTA. The incidence of RPE disruption was significant higher in PIC+CNV eyes when compared with those in PIC eyes (*P*=0.001, Table 1). All patients showed evidence of homogenous or heterogenous material in the macular fovea on SD-OCT images. PIC+CNV eyes had sub-RPE hyper-reflective materials, while the location of bulging homogenous hyper- or hypo-reflective material were found above the intact RPE in the PIC eyes.

**Table 1:** The distribution of RPE disruption and correlation analysis with PIC+CNV at different time points

	Baseline	4w	8w	12w
RPE disruption [n/N(%)]	0	5/18 (27.8)	5/18 (27.8)	5/18 (27.8)
PIC+CNV [n/N(%)]	0	5/5 (100)	5/5 (100)	5/5 (100)
PIC [n/N(%)]		0/13 (0)	0/13 (0)	0/13 (0)
*P* value		0.001	0.001	0.001

*RPE* retinal pigment epithelium, *PIC* punctate inner choroidopathy, *CNV* neovascularization, P*:* Compare the occurrence rate of RPE disruption on OCT with the incidence of PIC+CNV using Fisher's exact test

#### Choroidal transmission

At 4 weeks follow-up, 5 eyes had choroidal hyper-transmission and CNV, of which 2 eyes had a choroidal hypo-transmission component adjoined to hyper-transmission zone. After 8 weeks follow-up, hypo-transmission was also observed in the other 3 eyes. Lesions with hyper-transmission had a higher risk for developing CNV compared with those without hyper-transmission (*P*=1.17×10-3) during follow-up (Table 2). The incidence of choroidal hypo-transmission was significantly higher in PIC+CNV eyes than PIC eyes after 8 weeks (*P*=0.001, 0.001) (Table 3).

**Table 2:** The distribution of choroidal hyper-transmission and correlation analysis with PIC+CNV at different time points

	Baseline	4w	8w	12e
Hyper [n/N(%)]	0	5/18 (27.8)	5/18 (27.8)	5/18 (27.8)
PIC+CNV [n/N(%)]	0	5/5 (100)	5/5 (100)	5/5 (100)
PIC [n/N(%)]		0/13 (0)	0/13 (0)	0/13 (0)
*P* value		0.001	0.001	0.001

*Hyper* Hyper-transmission, *PIC* punctate inner choroidopathy, *CNV* neovascularization

*P:* Compare the occurrence rate of Hyper-transmission on OCT with the incidence of PIC+CNV using Fisher's exact test

**Table 3:** The distribution of choroidal hypo-transmission and correlation analysis with PIC+CNV at different time points

	Baseline	4w	8w	12e
Hypo [n/N(%)]	0	2/18 (11.1)	5/18 (27.8)	5/18 (27.8)
PIC+CNV [n/N(%)]	0	2/5 (40)	5/5 (100)	5/5 (100)
PIC [n/N(%)]		0/13 (0)	0/13 (0)	0/13 (0)
*P* value		0.065	0.001	0.001

*Hypo* Hypo-transmission, *PIC* punctate inner choroidopathy, *CNV* neovascularization

*P:* Compare the occurrence rate of Hypo-transmission on OCT with the incidence of PIC+CNV using Fisher's exact test

#### Damage of EZ

Ellipsoid zone (EZ) recovery began  at 4 weeks, and the recovery rate increased with time, from (5/18) 27.8% at 4 weeks to (8/18) 44.4% at 8 weeks, and about (9/18) half of EZ recovered at 12 weeks. There was no significant difference in the incidence of EZ damage between PIC+CNV and PIC patients (P=0.150, 0.196, 0.353) (Table 4).

**Table 4:** The distribution of EZ damage and correlation analysis with PIC+CNV at different time points

	Baseline	4w	8w	12w
EZ damage [n/N(%)]	18/18 (100)	13/18 (72.2)	10/18 (55.6)	9/18 (50)
PIC+CNV [n/N(%)]	0	5/5 (100)	4/5 (80)	3/5 (60)
PIC [n/N(%)]		8/13 (61.5)	6/13 (46.2)	6/13 (46.2)
*P*		0.150	0.196	0.353

*RPE* retinal pigment epithelium, *PIC* punctate inner choroidopathy, *EZ* ellipsoid zone

*P:* Compare the incidence rate of EZ damage on OCT with the incidence of PIC+CNV using Fisher's exact test

## Clinical examples

### Case 1

**Fig. 1:**
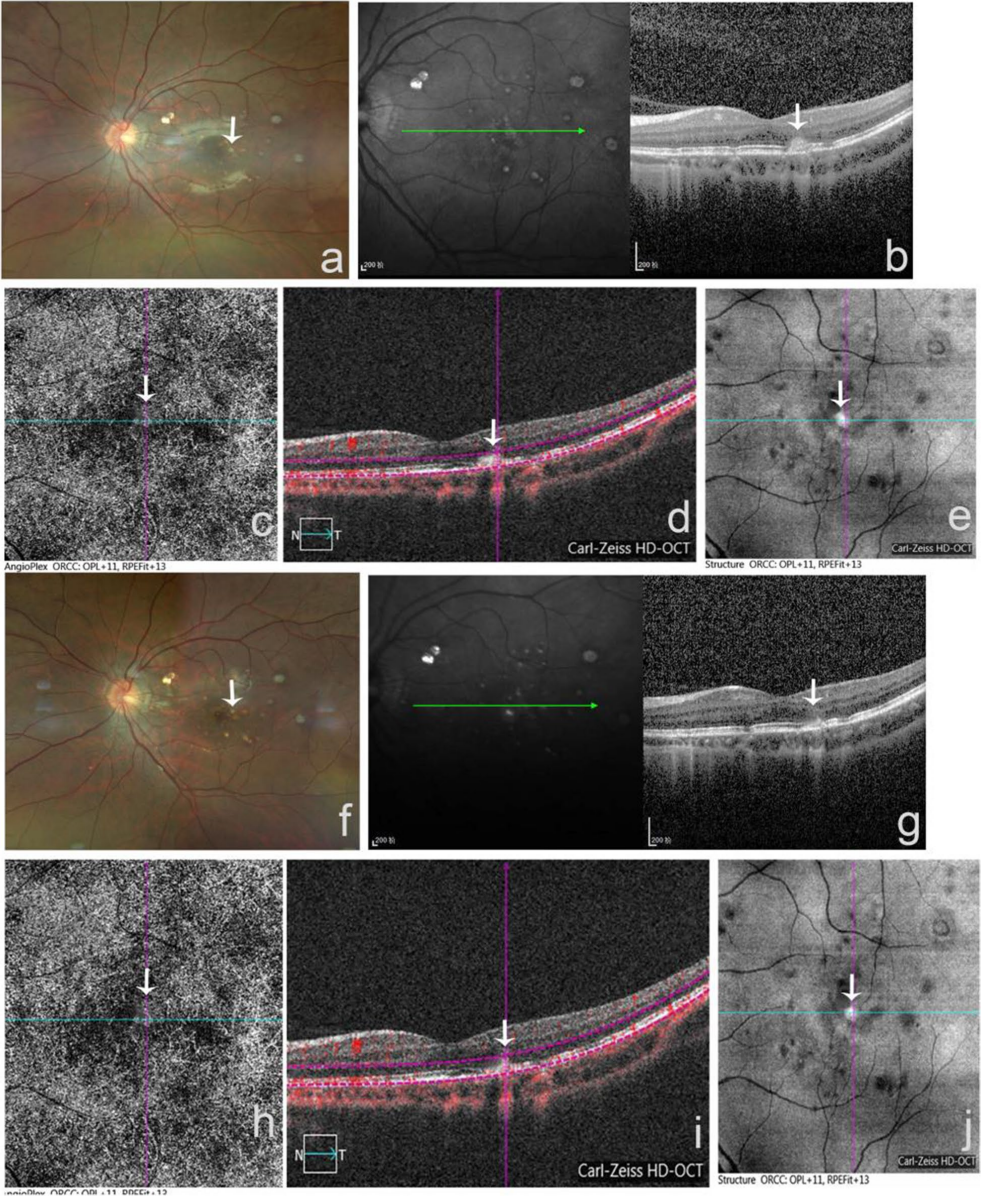
The fundus structure change of a patient had PIC without CNV. (**a**) Color fundus photography showed sporadic, small, round, yellow-white spots limited to the posterior pole of left eye and no hemorrhage (white arrow). (**b**) SD-OCT showed the presence of multiple punctured lesions around the macular fovea, and the EZ band of the lesion temporal to the fovea was blurred with hyper-reflective nodule (white arrow). RPE and BM were integral and neither choroidal hyper-transmission nor hypo-transmission was observed below the lesion. (**c**, **d**) OCTA angiography en face and B-scan showed no sign of neovascularization around the hyper-reflective nodule (white arrow). (**e**) OCTA structure en face showed a round-like, elevated lesion in the macular fovea (white arrow), surrounded with multiple punctured lesions with different sizes. (**f**) Twelve weeks later, the punctate elevated lesion in the macular fovea shrunk (white arrow). (**g**) SD-OCT showed hyper-reflective material under EZ shrunk and choroid maintained normal transmission (white arrow). (**h**, **i**) OCTA angio-Plex and B-scan showed no sign of CNV development during twelve weeks follow-up (white arrow). (**j**) OCTA structure en face showed a round-like, elevated lesion in the macular fovea (white arrow), which was smaller compared to (**e**)

A 26-year-old female complained of vision loss and metamorphopsia of her left eye with five days of evolution. The refraction was -7.25 D in both eyes. The best-corrected visual acuity (BCVA) in the right eye (RE) was 20/20 (6/6 in Snellen visual acuity of 6 meters) and in the left eye (LE) was 20/40 (6/12 in Snellen visual acuity of 6 meters). There were no signs of inflammation in the anterior chamber or vitreous cavity. Fundoscopy showed sporadic, small, round, yellow-white spots limited to the posterior pole of the left eye and revealed no hemorrhage (Fig. 1a). SD-OCT scanned the retinal white lesions in the temporal fovea and showed the presence of focal elevation of the EZ band with underlying hyper-reflective material. RPE and BM were integral and neither choroidal hyper-transmission nor hypo-transmission was observed below the lesion (Fig. 1b). No sign of CNV was found using OCTA (Fig. 1c, 1d, 1e). We diagnosed the patient with punctate inner choroidopathy without CNV, and regular follow-up visits were scheduled. Twelve weeks later, SD-OCT showed hyper-reflective material under EZ shrunk and choroid maintained normal transmission (Fig. 1f-1g). OCTA showed no neovascularization. (Fig. 1h-1j). Her visual acuity was 20/25 (6/7.5 in Snellen visual acuity of 6 meters), and the blurred vision was alleviated without therapeutic intervention.

### Case 2

**Fig. 2:**
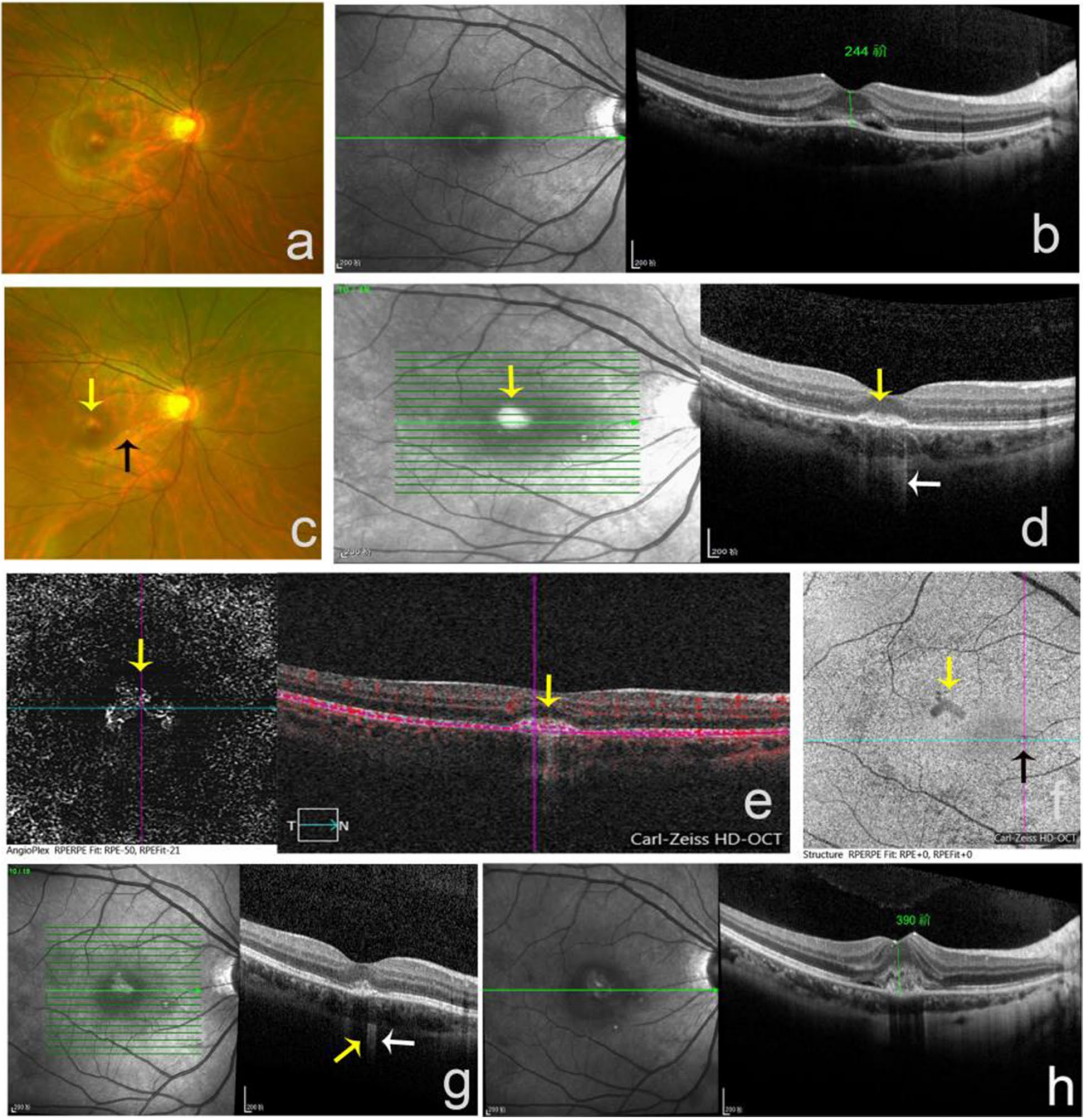
The funds structure of a patient had PIC without CNV at baseline and developed secondary CNV during follow-up. (**a**) At first visit, fundus photography showed a distributed yellow-white lesion in her macular region. (**b**) SD-OCT imaging revealed a disruption of the EZ zone, minimal sub-retinal fluid with a slight elevation of RPE, and normal choroidal transmission. (**c**) Two weeks later, fundus photography showed the lesion in the macular (yellow arrow) and a yellow-white spot appeared in the inferior nasal to the fovea (black arrow). (**d**) SD-OCT scanned the lesion in macular (yellow arrow) and showed moderate reflective material under RPE with a disruption of BM (yellow arrow), hyper-transmission could be found in choroid (white arrow). (**e**) Meanwhile, the 3×3 mm SD-OCTA angiography en face and b-scan images showed an abnormal vascular network (yellow arrow). (**f**) 6×6 mm SD-OCTA structure en face showed abnormal vascular network (yellow arrow) in the macular fovea and a dot inferior nasal to the fovea (black arrow). (**g**) Four weeks later, SD-OCT images began to show an area of choroidal hypo-transmission (yellow arrow) adjacent to the areas of a choroidal hyper-transmission (white arrow). (**h**) As the disease progressed, the vascular network grown larger and lesion extended

**Fig. 3:**
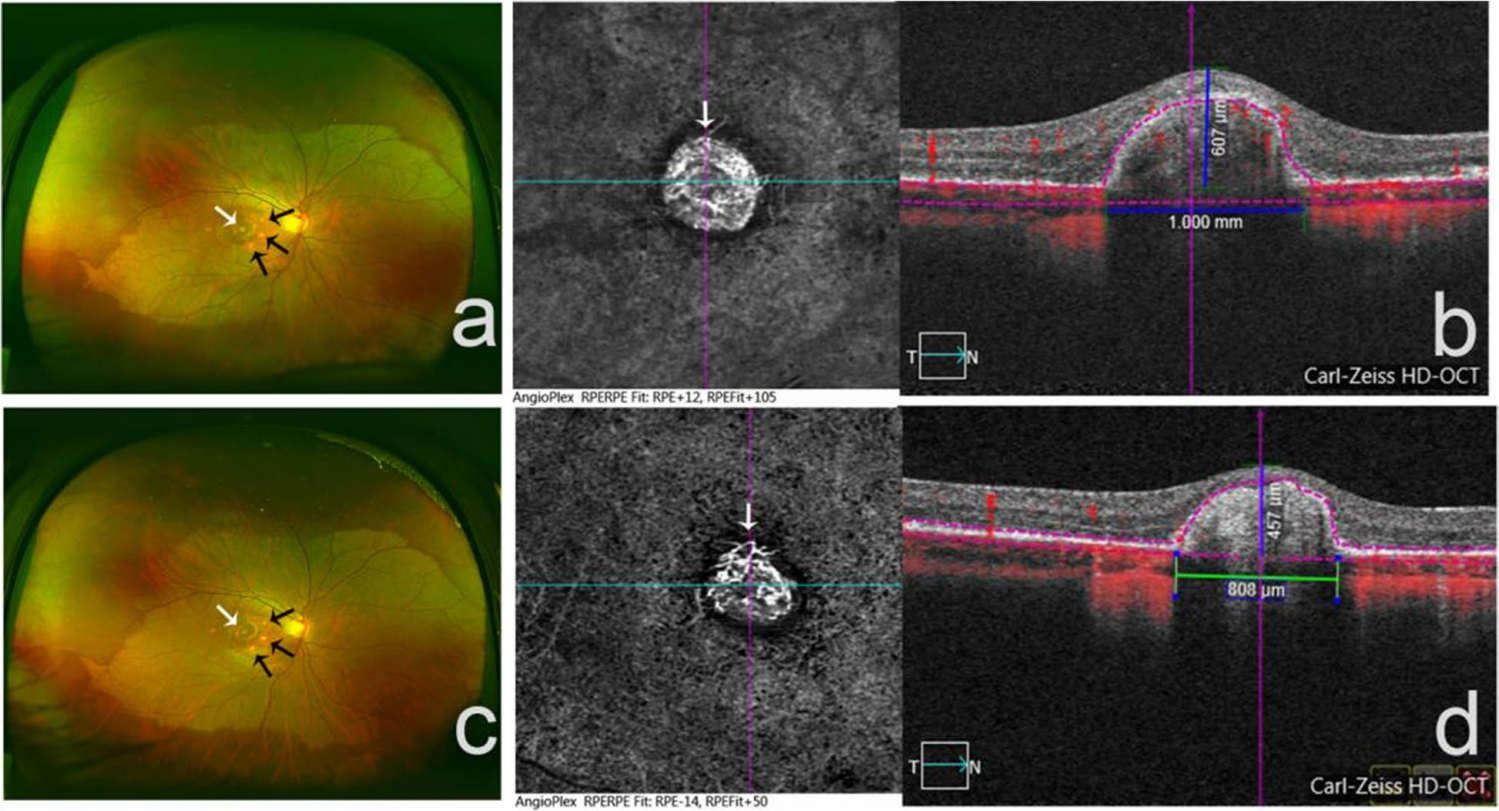
The PIC with secondary choroidal neovascularization before and after treatment. (**a**) fundus photography showed gray-white CNV lesion in the macular (white arrow), surrounded by three yellow-white dots (black arrows) (**b**) before treatment, OCTA showed vascular network (white arrow) and the cross-section of CNV in the retina (height and width of lesions were marked) (**c**) After anti-VEGF treatment, the fundus photography showed the CNV lesion in the macular (white arrow), surrounded with three dots (black arrows). (**d**) OCTA showed the neovascularization shrunk (white arrow) after the anti-VEGF treatment

A 22-year-old breast feeding woman was referred to our hospital with the complaint of metamorphopsia for two days in her right eye. She had myopia (-5.5 D) in both eyes. At the initial visit, her best-corrected visual acuity (BCVA) was 20/50 (6/15 in Snellen visual acuity of 6 meters) in the right eye and was 20/20 (6/6 in Snellen visual acuity of 6 meters) in the left eye. No remarkable changes were revealed in either ocular anterior segment by slit-lamp examination. Fundus photography showed a distributed yellow-white lesion in her macular region (Fig. 2a). SD-OCT imaging revealed disruption of the EZ zone with minimal sub-retinal fluid and a slight elevation of RPE, while no choroidal hyper-or hypo-transmission below the level of RPE was observed (Fig. 2b). A follow-up without any treatment was advised. Two weeks later, the patient came back with a complaint of decreased visual acuity, which was 20/200 (6/60 in Snellen visual acuity of 6 meters) in the right eye. A small yellow-white lesion appeared in the inferior nasal of fovea (Fig. 2c). SD-OCT imaging showed a moderate reflective material under RPE with disruption of BM (Bruch’s membrane), and hyper-transmission in the choroid (Fig. 2d). The 3×3 mm SD-OCTA en face image showed an abnormal vascular network (Fig. 2e-2f). As the disease progressed, SD-OCT images began to show an area of choroidal hypo-transmission adjacent to the areas of a choroidal hyper-transmission (Fig. 2g). The patient was diagnosed with CNV secondary to PIC. We advised the patient to receive anti-VEGF treatment, but she refused, which made the deterioration of the disease (Fig. 2h). Finally, the patient agreed to receive anti-VEGF, and her BCVA improved to 20/125 (6/38 in Snellen visual acuity of 6 meters), accompanied with the shrinkage of the neovascularization (Fig. 3).

## Discussion

Previous studies suggested the incidences of inflammatory CNV are ranged from 56.3% to 75% [5–8], and the incidences may increase over time [9] with repeated episodes of PIC. As a common complication of PIC, secondary CNV has similar features to inflammation lesions. They both present symptoms of vision loss, scotomata, or metamorphopsia, cause infiltrating lesions and show heterogeneous material in the outer retina and subretinal space. Since hyper-fluorescent and leakage can be found in both active inflammatory lesions and CNV, it is difficult to distinguish them through FFA. OCTA could significantly improve the detection rate, identify CNV from inflammatory lesions by catching abnormal blood flow signs in different layers of the retina [3, 10–12]. However, OCTA are not widely used than OCT in rural and community hospitals. To find a routine and available instrument to help distinguish the PIC inflammatory lesion from secondary CNV, we characterized SD-OCT features of PIC patients with clinical symptoms and tracked the changes during patients’ follow-up. We reported 17 patients (18 eyes) had recent acute blurring of vision and diagnosed with PIC, during 3 months of follow-up, five eyes (27.8%) developed PIC+CNV which were confirmed by OCTA within four weeks in a natural course. We found that the changes of microstructure on SD-OCT images could provide valuable information for distinguishing inflammatory lesions and CNV, especially the choroidal transmission and the microstructure of the outer retinal layer, RPE and BM.

Channa et al. [1] and Watzke et al. [13] described that clinically active patients would have RPE elevation with sub-RPE abnormal signals. Amer et al. [14] found that half of the inflammatory lesions were confined between an intact BM and RPE; while all inflammatory CNV had associated fluid exudation, and signs of RPE and photoreceptor cell disruption. Our results are consistent with these published data [4]. We have comprehensively observed and tracked the morphological characteristics such as RPE breaking point using SD-OCT star scan and volume modes to prevent lesions from neglecting. We observed that a bulging homogenous hyper- or hypo-reflective material located above the intact RPE in the PIC eyes, while the presence of RPE disruption along with hyper-reflective sub-RPE deposits were detected in PIC+CNV eyes. The incidence of PIC+CNV was significantly higher in eyes with RPE disruption compared with eyes without RPE disruption, because inflammatory CNV usually growth from the sub-RPE space, through a breach in RPE, into either the subretinal space or the outer retinal layers. The sub-RPE hyper-reflective deposits were the result of aggregation of inflammatory cells [15, 16]. Most importantly, we have found that none of the eyes had choroidal hyper-transmission or RPE breakage at baseline, at 4 weeks follow-up, 5 eyes had RPE disruption along with a choroidal hyper-transmission band below the PRE and were confirmed as secondary CNV by OCTA. This study indicated a significant correlation between the incidence of PIC+CNV and the occurrence of RPE disruption combined with high transmission. RPE defects and choroidal hyperreflectivity on OCT are usually results of atrophic punched out lesions in PIC and can be found in two situations; the new concurrent CNV causes such atrophic changes earlier, or the older PIC lesions with atrophy are the places where a CNV net is developed. The patients enrolled in our study were all at preliminary diagnosis and had acute blurring of vision within 4 weeks. At baseline, we could only find bulged RPE without breakage. RPE defects and choroidal hyperreflectivity were detected when CNV lesion was developed, suggesting the presence of PIC lesions with RPE defects and hyper-transmission.

In the early stages of PIC with secondary CNV lesions, it is difficult to determine by OCTA whether a flow signal is caused by new vessels or is only an artefact due to the relatively high reflectivity of the lesions. Thus, it cannot rule out the CNV that was not picked up entirely by OCTA in clinical diagnosis. In this study, we observed that at the first onset of PIC, RPE elevation without breakage could be only found in patients without CNV at 4 weeks, while CNV was detected in 100% of patients once the RPE disruption and hyper-transmission were visible. Therefore, these morphological changes could be the early signs of CNV which are easily identified on SD-OCT. There were also some previous researchers considered that the presence of choroidal hyper-transmission because of losing melanin granules, due to disruption of the RPE, photoreceptors and choroidal melanocytes in acute inflamed lesions [17]. Pachydaki SI et al. [18] reported a histopathological and electron microscopic analysis of excised concurrent CNV in PIC, demonstrated it contained of inner choroidal inflammatory cells which was composed of mature small lymphocytes [14]. Therefore, inflammation may be one of the etiology of concurrent CNV. As the secondary inflammatory CNV progressed, a choroidal hypo-transmission component could be detected in the middle of hyper-transmission zone, whereas this change could not be found in inflammatory lesions. In our study, the significant association between hypo-transmission and PIC+CNV was presented after 8 weeks. As we all known, materials that have high light absorptivity and reflectivity such as hemorrhage, pigment, or scars will lead to a shadow of the signals below on OCT images. These pathological changes in CNV complex resulted in an area of choroidal hypo-transmission associated with the lesion, which can be found in both myopic CNV (mCNV) and advanced PIC+CNV. However, due to the different pathogenesis, mCNV had only choroidal hypo-transmission and absence of hyper-transmission through all the course [4]. Therefore, the mixed lesion with hyper-transmission adjacent to hypo-transmission can be a sign to distinguish PIC+CNV from mCNV and inflammatory lesions. It is worth noting that the changes of choroidal transmission should be in contrast to the surrounding normal choroidal tissue but not to the hypo-intensity area. And under prompt treatment, some cases can remain in the stage of hyper-transmission.

In this study, all ellipsoid zone (EZ) bands had defects at baseline. At 4 weeks follow-up, we found that the defects of EZ including elevation or disruption started to recovered, and the recovery rate increased with time from 27.8% at 4 weeks to 44.4% at 8 weeks, and about half of EZ recovered at 12 weeks. The restoration of the ellipsoid zone has been previously noted in PIC and in other inflammatory conditions [1]. Spaide RF et al. [19] considered that the loss of EZ bands improved over time and was corresponding to the improvement of visual function. Our results were consisted with this conclusion. In this study, we have found EZ recovered along with vision improvement, which was observed in eyes without outer retinal atrophic changes or in eyes with significantly shrinkage of CNV after prompt treatment; while the eyes which developed new secondary CNV or had progressive CNV with extensive fibrosis showed persistent EZ disruption. Therefore, we think that the restoration of EZ was highly relative to the stage of disease and the degree of damage.

In conclusion, RPE disruption and choroidal hyper-transmission in SD-OCT are early signs of PIC+CNV, and SD-OCT is a useful tool for the differentiation and track of the progression of inflammatory lesions and inflammatory CNV in PIC. The increase of sampler size will greatly strengthen our study since a limited number of eyes were analyzed in this study. Future analysis of SD-OCT in patients with inflammatory diseases other than PIC may also provide more insight into the pathological processes and be helpful to monitor the prognosis of inflammatory CNV, which will enable us to better understand retinochoroid diagnosis and the effect of treatment.

## Data Availability

The datasets used and analyzed during the current study are available from the corresponding author on reasonable request.
